# A proposed approach for standardized reporting of data linkage processes and results.

**DOI:** 10.23889/ijpds.v7i3.1963

**Published:** 2022-08-25

**Authors:** Pooja Nair, Michael Smith, Maria Theochari

**Affiliations:** 1 NSW Health

## Objectives

Develop a digital solution for automated data ingestion and rapid update of the large-scale Human Services Dataset (HSDS) which brings together data from across government to take a powerful view of the service usage to improve outcomes of communities.

## Approach

The Centre for Health Record Linkage (CHeReL) hosts a secure, high-performing data linkage system, including a Master Linkage Key (MLK) of administrative health datasets, and generates linked data to inform policy decisions. Since 2018, CHeReL has also been annually linking over 70 frontline datasets to create a large-scale longitudinal linked dataset of over 2.5 billion records.

Over the course of 2021, the CHeReL led a project to incrementally improve the currency of the HSDS in compressed timeframes. This provided opportunity to assess value and feasibility of more frequent updates to the dataset within the evaluation and investment context.

## Results

The automated data Ingestion and validation led to a significant reduction in the data processing timeframes for the Accelerated linkage. We observed 80\% reduction in Data ingestion and 75\% reduction in data validation.

The digital solution also allows asset owners to register and approve new data providers, monitor their data provision in real-time and report on data sourcing. This provides transparency to the Asset Owner and reduces the need for time-intensive and manual processes to jointly monitor data provision with the Data Linkage Centre.

The digital solution also has the capability to support Data Providers automate their data feeds and provide on a regular basis through a secure non- touch process. This reduces on-going workload and ensures on-time provision.

## Conclusion

The process requires a systematic change in the upstream data source, and we requested participating agencies to send us data in an agreed format. The receipt of files in standard format is pivotal for reducing the overall timeframes of HSDS creation and leverage it for policy and investment purpose.

